# A Mixed-Method Approach to Explore Successful Recruitment and Treatment of Minority Patients on Therapeutic Cancer Clinical Trials at a Tertiary Referral Center Using Photo-Elicitation Interviews

**DOI:** 10.1089/heq.2023.0170

**Published:** 2024-02-28

**Authors:** Katharine A.R. Price, Rahma Warsame, Mary O'Shea, Yonghun Kim, Sara A. Ellingson, Gladys B. Asiedu

**Affiliations:** ^1^ Division of Medical Oncology, Mayo Clinic, Rochester, Minnesota, USA.; ^2^ Alix Mayo Medical School, Rochester, Minnesota, USA.; ^3^ Division of Hematology, Department of Medicine, Mayo Clinic, Rochester, Minnesota, USA.; ^4^ Mayo Clinic Center for the Science of Healthcare Delivery, Rochester, Minnesota, USA.

**Keywords:** underrepresented minority, cancer, photo-elicitation interview, clinical trial, barriers, facilitators

## Abstract

**Introduction:**

Under-represented minority patients (URM) enroll in cancer clinical trials (CCT) at low rates. To gain insight into barriers and facilitators to CCT enrollment, we conducted a mixed method study of URM patients who were successfully treated on a therapeutic CCT from 2018–2021 at all institutional sites.

**Methods:**

A retrospective chart review of 270 minority patients was conducted to identify patient demographics and characteristics. All living URM patients were requested to participate in a survey and qualitative interview using a photo elicitation technique.

**Results:**

Most patients who participated in a CCT were patients with solid tumors, metastatic disease, and did not live in a rural area. Survey data showed that the two most significant drivers of CCT enrollment were potential of benefit to self and to others (altruism). Direct recommendation from a healthcare provider to participate in CCT was critical. URM patients enrolled on a CCT experience a significant burden of symptoms and financial distress. Key themes identified from the interviews that motivated patients to participate included chance for cure, staying positive, altruism and advancement of science, and having diverse representation in research. Patient-level facilitators to participation included social support, cost coverage, and limited treatment options. Sytematic facilitators identified included minimizing logistical barriers, decentralizing cancer clinical trials, increasing awareness via patient narratives, diversifying research staff, minimizing cost, and being clear on puropose and benefit of the trial.

**Conclusion:**

Success stories of minority recruitment can provide useful information to enhance minority accrual. Photo elicitation interviews provide rich narratives of patient experience.

## Introduction

Clinical trials are critical for developing novel therapeutics and improving cancer-specific outcomes. Recent data suggest that patients in trials have better survival than trial eligible patients on standard therapy.^1,2^ Despite national efforts to improve recruitment, the percentage of underrepresented minority (URM) cancer patients treated on clinical trials has declined, underscoring that current strategies to increase URM accrual have been ineffective.^3^ URM patients have inferior cancer survival, and access to quality health care and novel agents is a known limiting factor.^4^ If our trial populations do not reflect the general population demographics, the applicability of results to URM patients may be jeopardized and health care disparities may increase further.

In 2019, there was a publication of best practices for inclusion of URM patients on cancer clinical trials (CCTs) at centers with a proven track record of URM patient recruitment.^5^ If these practices are to be successfully implemented, individual health care centers must understand the existing experiences of URM patients at their institution. Much has been published about barriers to research participation,^6–9^ yet we know little about the experiences, motivations, and decision-making of URM patients who were successfully treated on a cancer research study. Instead of focusing only on the negative influences on clinical trial enrollment, a more positive approach that acknowledges the existing strengths and everyday experiences of URM patients has the potential to allow a more nuanced view of the issue as it pertains specifically to our cancer populations.

Visual methods have been used in patient education and are becoming increasingly recognized as advantageous in health and illness research.^10–12^ The importance of visual research methods have been beneficial in constructing new, culturally tailored message concepts for underserved populations.^13,14^ In the wider context of an identified need for more efforts in trial accrual within URM populations, there is the potential to utilize visual data and narratives from URM patients in clinical trial recruitment and education programming. This study used a photo-elicitation interview (PEI) technique—a visual method tool—to explore and understand the motivations and facilitators of trial participation among URM patients. PEI is a powerful method for illuminating human experience and surfacing information, which may otherwise remain invisible or submerged in traditional research studies.^15^

Our rationale for using this approach was that information obtained through PEIs could potentially alter clinical trial recruitment efforts to include patient-generated visuals of URM trial experiences that enhance culturally tailored educational programs and could have long-term implications for future URM education surrounding clinical trials. In this study, we combined qualitative data from PEIs with quantitative data from the medical record and patient surveys to understand the experiences of minority patients treated on a CCT and to identify barriers and facilitators to clinical trial enrollment to inform future strategies toward clinical trial recruitment and community engagement. We chose to focus on interventional therapeutic cancer treatment trials (i.e., drug therapy trials) as such trials are considered higher risk and likely pose the most potential barriers to enrollment.

## Methods

### Patient recruitment and consent

Minority patients treated on an interventional therapeutic cancer treatment clinical trial at any Mayo Clinic site (Minnesota, Arizona, Florida) from 2018 to 2021 were identified through the electronic medical record and institutional clinical trial databases. All minority patients were included in the retrospective portion of the study (inclusive of Asian and more than one race). Living URM patients (defined as per National Institute of Health recommendations as black/African American, Native Hawaiian/Pacific Islander, Native American/Alaska Native, and Hispanic/Latino) were recruited for the survey and interview portion of the study. They were first approached via email and then with a follow-up phone call to invite them to participate in the survey and interview components of the study. Written consent was obtained by a member of the study staff. Participants were offered a token gift (value $50) for participation in the study.

This study was reviewed and approved by the Mayo Clinic Institutional Review Board.

### Quantitative-retrospective data collection

All subjects identified were eligible for the retrospective portion of the study. The following sociodemographic data were abstracted: patient age at the time of clinical trial enrollment, gender, race, ethnicity, preferred language, marital status, religion, insurance status (including type of insurance), location of home address (town or city, county, state, zip code), distance from home address to the treating site, and categorization of location of patient home address according to the U.S. Census rural–urban commuting area (RUCA) codes (isolated small rural town, small rural town, large rural city/town, or urban). The following cancer-specific information was abstracted as available: diagnosis (type of cancer), time from cancer diagnosis to start of treatment, stage at cancer diagnosis, treatment received (experimental or standard of care), treatment response, overall survival, and toxicity, including treatment-related hospitalizations or emergency room visits.

### Quantitative-prospective data collection

All living URM patients who agreed to participate were asked to complete a series of questionnaires and surveys to: (1) understand the patients' knowledge, attitude, and beliefs surrounding treatment on a CCT, including religious/spiritual beliefs, satisfaction with medical care, knowledge of cancer and clinical trials, reasons for participating in or refusing enrollment in a clinical trial, and how they heard about the research study, (2) assess their current financial distress using the validated Comprehensive Score of Financial Toxicity (COST) questionnaire, and (3) obtain detailed demographic information that could not be abstracted from the medical record, including education level, occupation, household income, number of household members, and availability of transportation. All three components of the survey were compiled into one survey booklet through the Mayo Clinic Survey Research Center (MCSRC) and either administered in person or mailed to the patient to complete and return.

### Qualitative-PEI

PEIs were conducted by a trained qualitative researcher with all the living participants who provided consent. The PEI technique involved semistructured interviews centered around photographs taken by the URM patients. The photographs were a symbolic representation of the clinical trial experience and reflected both concrete and abstract constructs, including motivations, experiences, and patient–provider communications that were considered meaningful and representative of the trial participation experience. Patients were given full autonomy to choose what they wanted to photograph. Interviews explored the URM patient experiences on a CCT, including aspects of care that went well, aspects of the process that could be improved, barriers they faced and how they were overcome (if they were overcome), and recommendations for improvement in the overall trial process.

Consented participants submitted at least two photographs via an email address created expressly for the purpose of this study and monitored only by study staff. Patient photographs were not shared with any other participant. Study staff printed the photographs and assigned each an identifying study number. Interviews were conducted over the phone or virtually. Each interview followed an interview guide and began with semistructured questions to understand the patient's experiences being treated in the setting of a clinical trial. For the photo-elicitation component, participants were asked to review the photographs for discussion. Each individually numbered photograph was then presented to the interviewee for discussion. The interviewee was asked to describe the content of their photograph, why the photograph was taken (its meaning, value, or symbolic representation to their trial participation), where it was taken, and if applicable, with whom the photograph was taken.

### Data analysis

For the quantitative components, descriptive statistics were used to summarize patient-level sociodemographics, cancer history and treatment information, and survey responses. All questionnaires were scored according to published scoring algorithms.^16^ Qualitative interviews were recorded, transcribed verbatim, deidentified, and the written transcripts checked for accuracy. Data from the PEIs were guided by a layered approach to ensure that the study team attended to the content of patient photographs in conjunction with the narrative produced by patients during the PEI. Using a “layered approach,” members of the research team independently read all the interview transcripts alongside patient-generated photographs and performed an inductive data analysis.

All transcripts and photographs were entered into the qualitative analysis software (NVivo 12 QSR International Pty Ltd.) for data management and analysis. This program allowed for coding, categorizing, and synthesis of data for specific ideas, themes, and topics of interest, and for generating reports at the end of the coding process.

## Results

### Quantitative data

Two hundred seventy URM patients treated on a therapeutic CCT from 2018 to 2021 were identified from the medical record. Retrospectively analyzed demographics and characteristics of patients with solid tumors (*n*=217) and hematologic malignancies (*n*=53) are summarized in Table 1. Of the 270 patients, 138 were alive (51.1%) at the time of data abstraction. Of the 138 living patients, 13 (9.4%) consented and participated prospectively in the interview and survey. All the survey results are summarized in Table 2.

**Table 1.** Retrospective Review of Characteristics of All Minority Patients with Solid Tumors and Hematologic Malignancies Enrolled on Therapeutic Cancer Clinical Trials at Mayo Clinic, 2019–2021

*N*=270	
Age (median)	58 Years, (range 2–88)
Gender	
Female	137 (50.7%)
Male	133 (49.3%)
Race	
Black/African American	70 (25.9%)
White	94 (34.8%)
American Indian/Alaskan Native	19 (7.1%)
Hawaiian/Pacific Islander	5 (1.9%)
Asian	58 (21.4%)
Unknown, not reported, or more than one race	24 (8.9%)
Ethnicity	
Hispanic	121 (44.9%)
Non-Hispanic	143 (52.9%)
Unknown or not reported	6 (2.2%)
Preferred language	
English	247 (91.5%)
Non-English	23 (8.5%)
Marital status	
Married	182 (67.4%)
Single	42 (15.5%)
Widowed	12 (4.4%)
Significant other/partner	5 (1.9%)
Divorced or separated	25 (9.3%)
Unknown or not reported	4 (1.5%)
Religion	
Roman Catholic	58 (21.5%)
Christian (all denominations)	73 (27.0%)
Islam/Muslim	2 (<1%)
Buddhist	6 (2.2%)
Hinduism	2 (<1%)
Native American spirituality	1 (<1%)
Agnostic	1 (<1%)
Nonspecific spiritual	2 (<1%)
Not religious	27 (10%)
Not reported	98 (36.3%)
Insurance status	
Government	52 (19.3%)
Private	142 (52.6%)
Private+Government	57 (21.1%)
Self-pay	3 (1.1%)
Unknown or not reported	9 (3.3%)
Not insured	7 (2.6%
RUCA code^a^	
Metropolitan (1–3)	230 (85.1%)
Micropolitan (4–6)	19 (7.0%)
Small town (7–9)	5 (1.9%)
Rural (10)	8 (3.0%)
Not reported	8 (3.0%)
Distance traveled to cancer center	
Median distance	93.6 Miles
Less than 20 miles	42 (15.5%)
20–50 Miles	62 (22.9%)
50–100 Miles	33 (12.2%)
Over 100 Miles	132 (48.8%)
Unknown	1 (<1%)
Type of clinical trial	
Phase I	80 (29.6%)
Phase I/II	38 (14.1%)
Phase II	73 (27.0%)
Phase II/III	3 (1.1%)
Phase III	52 (19.3%)
Phase IV	13 (4.8%)
Not reported	11 (4.1%)
Cancer stage at diagnosis (solid tumor only, *N*=217)	
Nonmetastatic	23 (10.5%)
Metastatic	104 (47.9%)
Not reported	90 (41.4%)
Tobacco-use status	
Never	168 (62.2%)
Former	76 (28.1%)
Current	14 (5.3%)
Not reported	12 (4.4%)

^a^ RUCA, rural–urban commuting area.

**Table 2.** Prospective Survey Results of Underrepresented Minority Patients Who Had Enrolled on Therapeutic Cancer Clinical Trials: Detailed Demographics, Cancer Beliefs, and Financial Toxicity

Patient demographics and characteristics (*N*=13)					
Gender identity					
Female	9 (69%)				
Male	4 (31%)				
Transgender	0 (0%)				
Do not identify as any of the above	0 (0%)				
Race					
Black or African American	3 (23%)				
American Indian or Alaska Native	2 (15%)				
Asian	2 (15%)				
Native Hawaiian or other Pacific Islander	1 (8%)				
White	1 (8%)				
Multiple races	3 (23%)				
Not answered	1 (8%)				
Hispanic or Latino origin or descent					
Yes	4 (30%)				
No	9 (70%)				
Highest grade or level of school completed					
8th Grade or less	0 (0%)				
Some high school, but did not graduate	0 (0%)				
High school graduate or General education diploma	2 (15%)				
Some college or 2-year degree	4 (30%)				
4-Year college graduate	2 (15%)				
More than 4-year college degree	5 (38%)				
Marital status					
Married	5 (38%)				
Living with partner	3 (23%)				
Divorced/separated	3 (23%)				
Widowed	1 (8%)				
Single	1 (8%)				
No. of people in household					
1	2 (15%)				
2	4 (30%)				
3	3 (23%)				
4	2 (15%)				
5	1 (8%)				
>5	1 (8%)				
No. of dependents					
0	9 (69%)				
1	1 (8%)				
2	1 (8%)				
3	1 (8%)				
>3	0 (0%)				
Mode of transportation to medical appointments					
Personal vehicle	9 (69%)				
Walk	0 (0%)				
Bike	0 (0%)				
Friend or family vehicle	1 (8%)				
Subsidized transportation	2 (15%)				
Public transportation	0 (0%)				
Difficulty finding transportation					
Yes	2 (15%)				
No	11 (85%)				
Combined annual income (pretax)					
Less than $25,000	3 (23%)				
$25,000 to less than $35,000	4 (30%)				
$35,000 to less than $50,000	1 (8%)				
$50,000 to less than $75,000	1 (8%)				
$75,000 or more	4 (30%)				
Type of health insurance					
Self-pay (none)	1 (8%)				
Private/employee provided	6 (46%)				
Medicare	4 (30%)				
Medicaid	1 (8%)				
Supplemental	1 (8%)				
Indian Health Service	1 (8%)				
One patient had both Medicare and Supplemental					
Occupational status					
Self-employed	0 (0%)				
Employed	4 (30%)				
Unemployed	1 (8%)				
Homemaker/caregiver	0 (0%)				
Student	0 (0%)				
Retired	2 (15%)				
Disabled	6 (46%)				
Receive support from religious faith					
Yes	4 (30%)				
Emotional	4				
Financial	0				
Informational	0				
No	9 (70%)				

Patient beliefs about cancer	Strongly disagree (%)	Somewhat disagree (%)	Neither agree/disagree (%)	Somewhat agree (%)	Strongly agree (%)
Cancer is an uncontrolled growth of cells.	0	7.7	15.4	30.8	46.2
It is necessary to have chemotherapy, or surgery, or radiation to cure cancer.	7.7	30.8	0	23.1	38.5
You can get cancer if someone puts a spell on you.	76.9	7.7	0	7.7	7.7
God will determine whether or not I will die from my cancer.	30.8	0	30.8	7.7	30.8
If a person worries about their cancer, it will get worse.	23.1	7.7	15.4	38.5	15.4

Financial assessment (COST survey)	Not at all (%)	A little bit (%)	Somewhat (%)	Quite a bit (%)	Very much (%)
I know I have enough money in savings, retirement, or assets to cover the costs of my treatment.	30.8	0	30.8	15.4	23.1
My out-of-pocket medical expenses are more than I thought they would be.	30.8	0	23.1	15.4	30.8
I worry about the financial problems I will have in the future as a result of my illness or treatment.	15.4	23.1	15.4	7.7	38.5
I feel I have no choice about the amount of money I spend on care.	15.4	0	15.4	30.8	38.5
I am frustrated that I cannot work or contribute as much as I usually do.	23.1	15.4	15.4	7.7	38.5
I am satisfied with my current financial situation.	30.8	23.1	7.7	7.7	30.8
I am able to meet my monthly expenses.	7.7	15.4	15.4	30.8	30.8
I feel financially stressed.	23.1	30.8	15.4	15.4	15.4
I am concerned about keeping my job and income, including work at home.	38.5	15.4	15.4	15.4	15.4
My cancer or treatment has reduced my satisfaction with my present financial situation.	15.4	30.8	15.4	0	38.5
I feel in control of my financial situation.^a^	16.7	25.0	33.3	8.3	16.7
My illness has been a financial hardship to my family and me.	23.1	30.8	0	0	46.2

^a^ One missing value (*N*=12).

COST, Comprehensive Score of Financial Toxicity.

### Survey results: detailed demographic data

Key findings from the detailed demographic data survey are as follows: Of the survey respondents, 69% were female, 23% each were black/African American or multiple races, 15% each were American Indian/Alaska Native or Asian, and 8% each were Native Hawaiian/Pacific Islander, white, or did not respond. Thirty percent of survey respondents were Hispanic or Latino. All survey respondents had at least a high school diploma or general education diploma; 85% had some amount of college education or higher. Most respondents had health insurance and did not have difficulty finding transportation to appointments. Forty-six percent of survey respondents were disabled, 30% employed, and 15% retired.

### Survey results: knowledge, attitudes, beliefs about cancer, and trial participation

General beliefs surrounding cancer are summarized in Table 2. All patients had heard of a clinical trial and 6 (46%) had known other clinical trial participants. On a scale of 0–10 (0=not important, 10=very important), the following factors influenced patients' decision to participate in a clinical trial from most to least important: chance that patient would benefit from the trial (mean 9.8), chance that trial participation would benefit others (9.7), progression of their cancer (9.6), no other treatment options available (9.3), out-of-pocket costs of standard therapy (8.9), advice from a physician (8.8), side effects of the experimental treatment (8.1), distance to the clinic/hospital (6.3), advice from family and friends (6.2), and religious or spiritual beliefs (4.7). All survey respondents had previously heard about clinical trials through direct communication from a health care provider; this was the most common way of hearing about a clinical trial.

Other means of learning about clinical trials included electronic communication from a health care provider (15.4%), the internet (15.4%), written communication (7.7%), and word of mouth (7.7%). No patients heard about clinical trials through social media. Nine patients (69%) had trust or strong trust in clinical trials or experimental therapy; four patients (30%) were neutral. No survey respondents indicated mistrust of clinical trials or experimental treatments.

### Survey results: financial toxicity

Table 2 summarizes the results of the COST survey for survey participants. Overall, the results demonstrate that many participants experience financial toxicity as part of cancer treatment. A total of 46.2% of respondents marked “very much” that their illness has been a financial hardship. The following percentages refer to the total percent of answers marked as “very much” and “quite a bit”: 30.8% of respondents had significant concern about keeping their job and income and 30.8% reported feeling significant financial stress. Only 38.5% of respondents were confident that they had enough savings to cover the cost of medical treatment, and 69.3% felt they had no choice about the amount of money they spend on care.

A total of 46.2% had significant worry about future financial trouble related to their illness or treatment and only 25% reported feeling in control of their financial situation. In response to a question about being able to meet monthly expense, 23.1% marked “not at all.”

### Qualitative results: PEI

After 13 interviews, thematic saturation defined as “no new ideas or themes emerging” was reached. Key themes about patients' motivation to participate in a CCT were chance for cure, staying positive, giving back to others, having a representation in research, and advancing science. Key themes about facilitators to participation were as follows: supportive family, cost coverage for treatment, and limited treatment options. Key themes on participation were as follows: minimizing logistical barriers, decentralizing CCTs, increasing awareness via patient narratives, diversifying research staff, minimizing cost, and being clear on purpose and benefit of the trial. Representative patient quotes supporting the key themes relating to barriers and facilitators to trial enrollment are summarized in Table 3.

**Table 3.** Key Themes and Representative Quotes from Photo-Elicitation Interviews of Minority Patients Treated on a Cancer Clinical Trial

Thematic category	Example quotes
Patients' motivations for participating in clinical trials (factors that encourage participation) Cure Staying positive Giving back (altruism) Representation Advance research and science	I was excited because I figured maybe it was something that could cure me. So I never turned down a trial. Pt1 I always was looking for an opportunity to give back in some way because I had learned along my journey that there are a lot of folks that have volunteered in so many different ways and that has impacted my treatment … that's our way of giving back. Pt 2 Well, let's put it this way. I don't think I would have as much success if there wasn't somebody before me doing the trial. That's why I am where I am, is because of somebody else … Things are better for me because somebody before me took part in the trial. I would just hope that people would realize that it's a good thing to do for the next person that has to go through this, to take part in clinical trials. Pt4 I feel like definitely there are not enough minority groups, African-Americans … like we're so underrepresented in so many of these trials … And if we don't put ourselves out there, then we're leaving ourselves at a huge disadvantage of being helped when we need the help if we do come across a point in our lives where we have a particular disease or cancer, whether it be cancer or any other ailment. I feel that there's a huge need for us to be more represented. Pt 5 If science develops a drug with minimal side effects, that'll either suppress or get rid of the cancer and they need somebody to test it. I'm, I'm definitely all for that. And if it works … that's a plus. P7
Facilitators to clinical trial participation (factors that makes it easy to participate) Supportive family Cost coverage Limited options	My husband said, I'll adjust my office meeting, signing, whatever let's do this. This is really good and let's do that …We just thought about it and the logistics piece was something that I had to rely on him because I cannot drive okay. Post treatment. That was the only thing. Pt 2 So, I can't buy a life insurance policy anymore, and changing jobs might make it so that I can't get health insurance and all of those things. So, the positive sides to that with being in a study is the cost of the drug is covered. and the doctor visits that are associated with the study are covered, and that definitely takes a weight off of your shoulders. Pt5 Whether it's a study drug or a drug that I have to pay for, I want what's going to work best for me. And the other thing is with my cancer, there aren't that many drugs available. We're very limited in our choices. P5
Opportunities and considerations for increasing minority patients CT participation Education, information availability, awareness, using patient narratives Minimizing barriers to CT Logistics Decentralizing trials Diversifying workforce and cultural concordance Clarifying the purpose and benefits of population characteristics during CT introduction and delivery Creating support groups and connecting patients on trials Cost and finances Outreach and advertisement	I don't think there's not a lot of information available … To talk about it and to talk about the benefits of it. Pt2 I think one would definitely be sharing firsthand account from whoever may have been on one before or is currently in a trial so they can share their experiences. Good, bad, ugly, whatever. Pt2 So, I think the frequency of the visits needs to be something that is taken into consideration for the patients. I understand that they want to monitor closely, but over time and as they're more progressed into the study, frequency can be adjusted to some extent, at least for some studies …. But I think if they work together with the patients' local physicians to counteract that worry and that concern, that would be helpful. Pt 5 You need to make sure that there's someone in that group, in that care team, that you know they feel comfortable with and someone that they trust. And that may mean having someone that looks like them give them that information. P5 And there are so many people … and they all seem to have the same perspective. “Well, are you actually receiving a medication? How do you know? Are you on a drug? They want you to think that you're on the drug.” … I think it's important to know how the documentation is going to be used. What is the purpose of the particular trial … and am I part of an initial study group? But I would appreciate knowing the demographics of the other people who are in the trial in a very general, broad, HIPAA way. P4 I've tried looking through different support group online to see if there are many people like me and they are few and far between to find anyone who's a minority, especially under the age of 50, I might as well be 50 at this point, but close to my age or close to my heritage and my background …, I would love to know if there were other people like me who were participating in the study and how they were responding to that. P6 There should be somewhere where you can tick a box to say, “Yes, I would love to get in touch with someone else who's part of this trial for some support.” Because other than that, there is no support for us in a trial. “Well, I want to speak to other people and find out for myself if they've really felt that.” So, I mean, that type of therapy, because to me that would be therapeutic, to meet with other people who share similar experiences. P5 I don't know if there's more assistance that could be done, either financial assistance or there's a way where if the person like myself, I had to fly and I have to get accommodation, where Mayo could work out with an airline or a hotel, Airbnb of the sort that can lessen the cost for the person on the clinical trial. Pt 9 You really don't know about clinical trials unless your doctors bring them up … So if Mayo or some other medical place put out a list, not a list, but I don't know, information about upcoming clinical trials or what you may be interested in.

**FIG. 1.**
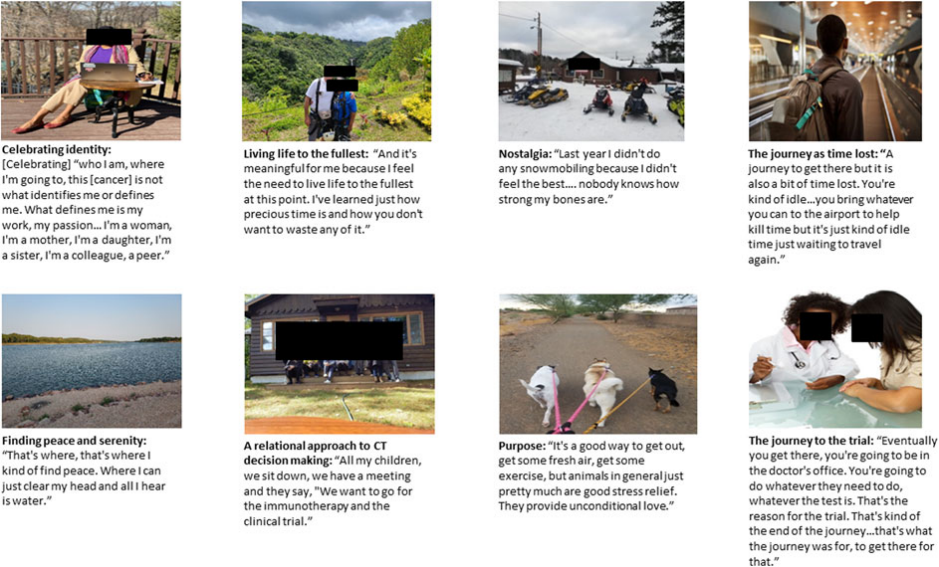
Underrepresented minority patient narratives and photographs from photo-elicitation interviews of cancer clinical trial experience.

Photographs showed symbolic representation of patients' general experiences around clinical trial, including celebrating their “new identity,” finding peace in moments of nostalgia, their journey to the trial, and the feeling of lost times and yet finding a new purpose to live through CT participation. Importantly, patients described decision-making in CT participation as a relational approach. Representative photographs from the interviews with patient narrative are shown in Figure 1.

## Discussion

In this mixed-method, single-institution study, we examine a population of minority patients who successfully enrolled in a therapeutic cancer clinic trial at a major academic center to gain insight into those patients who were able to overcome barriers to research participation and to learn from their experiences to inform future strategies for recruitment of diverse populations to CCTs. The patients who did *not* participate in a CCT are as informative as the demographics of our study population itself. Of the 270 patients making up the study population, only 4% were from rural areas and the majority spoke English as their preferred language. Most patients treated on trial had metastatic disease and 75% had solid tumor malignancies.

These study demographics highlight opportunities to better engage the patients not seen in this sample such as those living in rural areas and those with non-English as a preferred language, as well as opportunities to develop trials focusing on earlier stage disease and hematologic malignancies. The low consent rate obtained from living patients for the surveys and interviews also highlights the difficulty of connecting with this population of patients, even among a group that has already participated in clinical research. Ultimately the onus of creating meaningful relationships and engaging minority patients is on the health care system, institution, and researchers.

All participants in this study had health insurance of some kind further highlighting the difficulty of reaching those populations that are the most vulnerable and marginalized. More robust, community-engaged, and community-centered strategies to facilitate bidirectional communication between researchers and minority and rural communities are critical to lay the foundation for future research participation.

Several important lessons were learned from the survey and the interviews that could inform recruitment strategies moving forward. The two most important reasons why patients decided to participate in a clinical trial were the potential for benefit to themselves or to others. Helping future cancer patients appears to be a significant motivator to enroll in a CCT, and this message of altruism could be more deliberately incorporated into marketing strategies and individual physician–patient conversations to improve recruitment of diverse patients. Altruism as a potential driver of research study participation has been previously described.^17–19^

Prior studies investigating altruism have found that pure altruism—defined as “acting with an unselfish regard for others”—is difficult to separate from individuals' desire for self-benefit and may not be enough to overcome personal barriers to trial participation. As such, altruism could be a strategy to engage initial patient interest in research participation but needs to be backed up with additional resources such as transportation and financial assistance. Although this is the first study to highlight altruism as an independent variable for clinical trial participation in a minority population, altruism has been described as a key motivator to URM students to pursue a career in biomedical science.^20^

Several other potentially actionable themes were highlighted from the survey and interview results to improve CCT recruitment. We found that a direct recommendation to participate from a health care provider is critical to enrollment, speaking to the physician–patient relationship and to the importance of underlying trust as a motivator for research participation. Shared decision-making is a common practice in modern medicine but does not preclude a clinician from providing a strong treatment recommendation based on expertise and nuanced understanding of the individual patient's scenario. Provider education on the importance of systematically advocating for trial participation to all patients and a direct recommendation to participate in research could be helpful to increase trial enrollment and to counteract the known unconscious biases that can exist from health care providers toward minority patients when considering clinical trial enrollment.^21^

Our data demonstrate that cancer patients, even those who can participate in research, carry a significant burden of physical and emotional symptoms and financial distress that impact the quality of life. Assessing social determinants of health and harnessing institutional and governmental resources in a deliberate and systematic manner should be part of routine cancer care to try and mitigate patient burden and barriers to clinical trial enrollment and retention. Recognizing that treatment in the setting of a clinical trial results in significant out-of-pocket expenses beyond just the cost of the cancer care itself (transportation, childcare, loss of workdays, food) is critical and building patient remuneration into clinical trial budgets should be routine.

## Limitations of Study

The most significant limitation of the study is the overall small sample size and the subsequent low survey response rate. Accrual of URM patients to CCTs is low nationwide and has remained so despite the increased awareness and efforts. Barriers to the survey participation may include time, current health status given the cancer population, research participation burnout, and competing priorities. Data generalizability may be limited as this is a small single-institution study, but the data obtained, particularly the patient narratives, are rich and informative.

## Health Equity Implications

Examination of success stories of minority recruitment to therapeutic CCTs can provide useful information to develop strategies to enhance minority accrual. Qualitative PEIs of URM cancer patients who participated in a clinical trial are feasible and provide rich narratives of patient experience. Such an approach could be valuable on an institutional level to explore issues and perspectives unique to the population being served. A direct recommendation from a health care provider to participate in clinical research can be helpful to facilitate inclusive research practices. Understanding the URM cancer patient perspective and motivations such as the desire to help others through research can inform research recruitment strategies and patient outreach. Future directions for this work include evaluation of the impact of patient narratives and visuals in educational and community-facing programming surrounding cancer research on clinical trial recruitment.

## Abbreviations Used

CCT: cancer clinical trial
COST: Comprehensive Score of Financial Toxicity
PEI: photo-elicitation interview
RUCA: rural–urban commuting area
URM: underrepresented minority
